# Glucagon-Like Peptide 1 Receptor Agonists and Sodium–Glucose Cotransporter 2 Inhibitors Improve Renal Resistive Index in Patients With Type 2 Diabetes: A 26-Week Prospective Observational Real-Life Study

**DOI:** 10.1155/jdr/8182211

**Published:** 2025-02-10

**Authors:** Alfredo Vozza, Sara Volpe, Carlo Custodero, Valentina Colaianni, Valentina Lavarra, Domenico Triggiani, Lucilla Crudele, Alessandro Bergamasco, Gianfranco Antonica, Cosimo Tortorella, Giuseppina Piazzolla

**Affiliations:** Interdisciplinary Department of Medicine, School of Medicine, University of Bari “Aldo Moro”, Bari, Italy

**Keywords:** chronic kidney disease, diabetic kidney disease, glucagon-like peptide 1 receptor agonists, renal damage, renal Doppler ultrasound, renal hemodynamic, renal resistive index, sodium–glucose cotransporter 2 inhibitors, Type 2 diabetes

## Abstract

Diabetic kidney disease (DKD) is one of the most life-threatening complications of diabetes and a leading cause of chronic kidney disease. Glucagon-like peptide 1 receptor agonists (GLP1-RAs) or sodium–glucose cotransporter 2 inhibitors (SGLT2is) appear to improve renal outcome in patients with Type 2 diabetes (T2D). In this context, the renal resistive index (RRI) is a useful doppler measure to study DKD and predict its evolution. The aim of this work was to study the effect of treatment with GLP1-RA or SGLT2i on RRI and the relationship between RRI and glycometabolic parameters. One hundred forty-five patients with T2D were enrolled in the study and treated for 26 weeks with once-weekly GLP1-RA (38 patients with dulaglutide and 39 with semaglutide), SGLT2i (40 patients), or other therapies (28 control patients). Clinical, anthropometric, and hematochemical parameters and RRI were measured at baseline (T0) and after 6 months of treatment (T6). Changes at 6 months were studied and compared by treatment group. Patients were predominantly male (58.6%), overweight (93.0%) or frankly obese (60.0%), with hypertension (90.0%) and high (> 0.64) or pathological (> 0.7) RRI values (82.0% or 37.0%, respectively). At baseline, RRI correlated positively with age, fasting blood glucose, glycated hemoglobin (HbA1c), triglycerides, and albuminuria and negatively with estimated-glomerular filtration rate (e-GFR). At T6, patients treated with either GLP1-RA or SGLT2i showed a significant improvement in RRI but not in albuminuria or e-GFR, compared with homologous at baseline. In particular, RRI normalized in 32% and 30% of patients on therapy with GLP1-RA and SGLT2i, respectively, while remaining almost unchanged in controls. Notably, the RRI improvement was independent of age, gender, diabetes duration, and changes in BMI, waist circumference, HbA1c, and e-GFR. In conclusion, RRI can be used to detect early kidney damage and follow the evolution of DKD. GLP1-RA and SGLT2i improve RRI, demonstrating benefits on cardiovascular risk and renal outcomes.

## 1. Introduction

Type 2 diabetes (T2D) is a metabolic disorder characterized by impaired glucose homeostasis and insulin resistance. The natural course of the disease worsens with the onset of micro- and macrovascular complications, including nephropathy [1].

The mechanisms underlying the development of diabetic nephropathy are multiple and mainly related to sclerosis of intra- and extrarenal arterial vessels, microangiopathy of glomerular capillaries, and afferent and efferent arteriole changes as well as interstitial and tubular alterations.

Diabetic microvascular complications are often asymptomatic and moderately progressive during the early stages. Therefore, to be able to conduct early screening and follow-up of kidney hemodynamics with a safe and noninvasive tool is paramount.

The renal resistive index (RRI) is a semiquantitative ultrasonographic (US) Doppler parameter of flow velocities in intraparenchymal renal arteries, detected by a simple and repeatable method. RRI is essentially an expression of tissue resistances downstream of the sampled vessel, since it integrates arteriolar compliance, pulsatility, and microcirculation impedance. It is therefore closely related to renal atherosclerosis [2].

Several studies have described a close relationship between RRI and diabetic nephropathy. Indeed, RRI has been shown to discriminate diabetic kidney disease (DKD) from other forms of nephropathy [3], to predict the evolution of kidney disease [4] and to be correlated directly with the degree of nephropathy [2, 5].

However, data in the literature on the relationship between RRI, microalbuminuria, glycated hemoglobin (HbA1c), cholesterol, and estimated-glomerular filtration rate (e-GFR) are conflicting, and further studies are needed [2, 4–7].

New antidiabetic drugs, belonging to the class of glucagon-like peptide 1 receptor agonists (GLP1-RAs) or sodium–glucose cotransporter 2 inhibitors (SGLT2is), although acting through profoundly different mechanisms, appear to improve the renal outcome in patients with T2D [8–10]. SGLT2is have been shown to achieve this goal independently of glycemic control, so that dapagliflozin first and empagliflozin shortly thereafter have recently gained therapeutic indication for chronic kidney disease (CKD) even in nondiabetic patients [11, 12]. Experimental studies have also demonstrated the potential of SGLT2i in improving endothelial function, arterial stiffness, and RRI [13, 14].

With regard to the direct nephroprotective effects of GLP1-RA, the available data have so far mostly been extrapolated from studies with primary cardiovascular (CV) outcomes [15, 16], but very recently, the FLOW trial reported a clear benefit of semaglutide on renal outcomes and death from CV causes in patients with T2D and CKD [17].

However, to our knowledge, there are no studies specifically evaluating the effects of once-weekly GLP1-RA on RRI.

The primary aim of the study was to evaluate, in a population of patients with T2D, the effect of a 26-week course of GLP1-RA or SGLT2i therapy on RRI. The relationship between RRI and anthropometric parameters (BMI and waist circumference), glycometabolic profile, and renal function, before and after therapy with both classes of antidiabetic drugs, was also studied.

## 2. Materials and Methods

### 2.1. Study Design

This prospective, observational, real-life study was carried out in the Metabolic Disorders Outpatients Clinic of the Department of Internal Medicine at the University of Bari, Italy, from June 2022 to December 2022. The study was conducted in accordance with the general ethical principles for medical research involving human subjects of the Declaration of Helsinki [18]. The study protocol was formally approved by the Clinical Investigation Ethics Committee of the University of Bari (ID: PZZ_DM2 2020, Number 6468, Version 2, 14 September 2020).

In order to proceed with enrollment, patients had to be older than 18 years and able to provide informed consent to participate in the study. First, they had to be patients with T2D, whether the diagnosis had already been established or formulated at the time of our clinical observation. Overall, 200 patients were examined. After a complete evaluation of the clinical, biohumoral, and instrumental data, they had to be eligible for treatment of diabetic disease with drugs belonging to the class of once-weekly GLP1-RA (semaglutide or dulaglutide) or SGLT2i (canagliflozin, dapagliflozin, empagliflozin). Only patients who completed at least 6 months of treatment and underwent the half-year visit were included in the statistical analysis. Patients with Type 1 diabetes, malabsorption disease, chronic pancreatitis, active or advanced neoplastic disease, kidney stones, glomerulopathies, renal artery stenosis, interstitial kidney disease, and uncontrolled hypertension were excluded from the study to eliminate confounding factors in the measurements. Patients already taking a drug belonging to one of the pharmacological classes under study, pregnant or lactating patients, and those who did not give informed consent to participate in the study were also excluded. Finally, the total number of patients enrolled was 145.

The overall follow-up period was 26 weeks. Visits were carried out at baseline (T0) and after 6 months of treatment (T6).

All patients were informed about the study protocol and the different treatment options, listing the potential side effects and how to administer each drug. Inclusion in the study was preceded by obtaining written informed consent.

Based on the cardiovascular risk (CVR) profile, cardiac and renal function, and preference to receive oral or subcutaneous therapy, patients were subdivided into two groups.

Group 1 (*n* = 77) included 42 male and 35 female subjects, who started treatment with semaglutide (*n* = 39) or dulaglutide (*n* = 38), administered subcutaneously weekly. Semaglutide was prescribed at the dose of 0.25 mg once weekly (qw) for 4 weeks and then raised to 0.5 mg qw. Similarly, dulaglutide was prescribed at 0.75 mg qw for the first 4 weeks and then raised to 1.5 mg qw.

Group 2 (*n* = 40) included 30 male and 10 female subjects, who started treatment with a SGLT2i: canagliflozin (*n* = 10, 100 mg/die), dapagliflozin (*n* = 20, 10 mg/die), or empagliflozin (*n* = 10, 10 mg/die) in daily oral administration.

It should be emphasized that the characteristics of the enrolled patients and the prescribed therapy closely reflect our daily clinical practice. Most of our patients have varying degrees of obesity and weight loss is one of the desired outcomes. This makes GLP1-RA, if indicated, more favorably accepted by patients.

Both groups were taking the maximum tolerated dose of metformin. Patients who refused both GLP1-RA and SGLT2i (due to mistrust of new therapies or concerns related to potential side effects) were assigned to the control group (*n* = 28, 13 males and 15 females) and received conventional therapy: 24 patients were prescribed metformin alone, 2 a combination of metformin + dipeptidyl peptidase 4 inhibitor (DPP4-i), and 2 a triple combination of metformin, DPP4-i, and insulin.

All patients were encouraged to increase their physical activity, to follow a written list of nutritional advice, and to improve their lifestyles.

### 2.2. Clinical and Laboratory Evaluation

All patients underwent an evaluation of clinical (general examination, blood pressure measurement, heart rate), anthropometric (BMI, waist circumference), and hematochemical (blood glucose, HbA1c, C-peptide, insulinemia, lipid balance, serum creatinine, and urinary albumin–creatinine ratio (UACR)—measured on morning spot urine) parameters immediately before inclusion in the study (T0) and after 6 months of therapy (T6).

### 2.3. RRI Measurement

Renal US scans were performed with the patient fasting for at least 8 h using a General Electric Logiq E9 ultrasound machine equipped with a high-resolution Convex probe with a frequency of 1–8 MHz. RRI was measured by sampling an interlobar (or arciform) artery at the superior polar, inferior polar, and mesorenal level. For each vessel sampled, three waves of overlapping size were evaluated and averaged. Then, for each kidney, the average of the three sampled vessels was calculated and the highest RI value between the two kidneys was used for statistical analysis.

The RRI was calculated by applying the formula peak systolic velocity − telediastolic velocity/peak systolic velocity.

RRI values were considered normal between 0.58 and 0.64 and frankly pathological above 0.70 [19].

### 2.4. Statistical Analysis

The descriptive statistics were expressed as absolute and relative (percent) frequencies for categorical variables and as mean and standard deviation for continuous variables. Normal distribution of continuous variables was checked by the Kolmogorov–Smirnov test as well as the visual inspection of the Q–Q plots. Parameters characterized by not normal distribution were expressed as median and interquartile range (IQR). When appropriate, variables that were not normally distributed were transformed into natural logarithms.

Paired-samples *t*-test stratified by individual antidiabetic treatments was performed to test whether there were significant changes in the parameters of interest between baseline and 6-month follow-up. Changes at 6 months in anthropometric, biohumoral, and renal US parameters were also compared by treatment group. Differences between groups were tested by analysis of variance (ANOVA) with Bonferroni correction for multiple comparisons for continuous variables and Pearson's *χ*^2^ test for categorical variables.

Pearson and Spearman correlation coefficients were calculated to test the association between the RRI and other variables of interest as well as between changes in the same parameters over 6 months of follow-up.

The McNemar test was used to test for differences in the frequencies of subjects with RRI > 0.64 between baseline and 6-month follow-up in each treatment group.

Linear mixed models with unstructured parameterization matrix for repeated measures were developed in order to estimate the change in time of RRI according to the antidiabetic treatment (Model 1: GLP1-RAs vs. SGLT2i vs. controls and Model 2: semaglutide vs. dulaglutide vs. controls; in both models, patients taking conventional therapy served as the control group) adjusting for potential confounding factors (i.e., age, gender, diabetes duration) and change of HbA1c, BMI, waist circumference, and e-GFR.

A two-tailed significance level of *p* = 0.05 was set for each test. Analyses were performed with SPSS v26.0 software for Windows (SPSS, Chicago, Illinois, United States).

## 3. Results

### 3.1. Descriptive Statistics of the Study Population

The baseline characteristics of the entire study population are described in Table 1.

Patients were predominantly male (58.6%), with a male:female ratio of 1.4:1, in line with the epidemiological data in the literature [20]. The duration of T2D was 4.5 years as median, ranging from 0 (new diagnosis) to 34 years. A large proportion of the patients had hypertension (90.0%) and was overweight (93.0%) or frankly obese (60.0%), with high (> 0.64) or pathological (> 0.7) RRI values (82.0% or 37.0%, respectively). In line with the short duration of diabetes, a very small percentage of patients had retinopathy or neuropathy (4.1% and 1.3%, respectively), with no differences between groups. Interestingly, the wide variability of values shown by the enrolled population, as regards some parameters such as age, BMI, waist circumference, glycemic profile, lipid balance, renal function, and RRI, is in agreement with the real life nature of the study.

**Figure 1:**
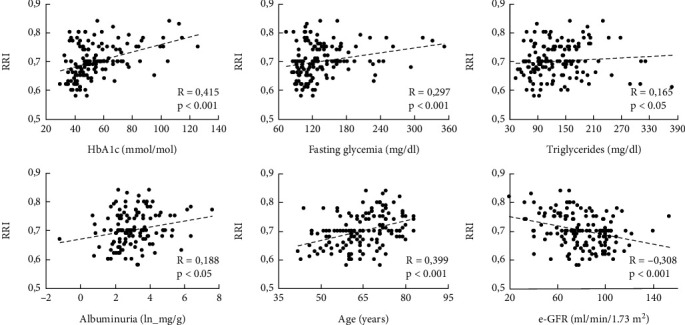
Bivariate correlations at baseline between RRI and anthropometric and biohumoral parameters in the study population (*n* = 145). Abbreviations: HbA1c: glycated hemoglobin; e-GFR: estimated-glomerular filtration rate; RRI: renal resistive index.

Before starting any therapy, the association between clinical and biochemical variables of interest and RRI was assessed by calculating Pearson and Spearman correlation coefficients at baseline in the entire study population. Using this approach, we found that RRI was correlated positively with age, fasting blood glycemia, HbA1c, triglycerides, and albuminuria and negatively with e-GFR (Figure 1), while no significant correlation was detected between RRI and BMI, waist circumference, or any cholesterol parameter.

Patients were subdivided into two groups according to the prescribed therapy: subcutaneous GLP1-RA (*n* = 77) or SGLT2i (*n* = 40). Twenty-eight patients who refused new antidiabetic drugs were included in the control group (*n* = 28). The characteristics of patients at T0 after subdivision into groups are schematized in Table 1.

Comparison between groups showed that the gender of patients and the percentages of hypertensive patients were equally distributed. All hypertensive patients were taking sartans or ACE inhibitors, and none required changes in antihypertensive therapy during the observation period.

Patients starting GLP1-RA treatment had a significantly higher BMI and waist circumference, while the SGLT2i group showed significantly higher fasting blood glucose and HbA1c than the other groups.

No significant differences were observed between groups in terms of e-GFR, albuminuria, C-peptide, and lipid profile, except for the presence of lower HDL values in the group starting GLP1-RA compared with controls.

Finally, RRI values were found to be significantly higher in the SGLT2i group compared with patients starting therapy with GLP1-RA.

### 3.2. Changes in Anthropometric, Biohumoral, and Renal Parameters in Groups of Patients With T2D Following a 26-Week Course of Therapy and Comparison Between Groups

Changes in anthropometric and biohumoral parameters after 6 months of follow-up in the GLP1-RA, SGLT2i, or control group are schematized in Table 2. Results of the statistical comparisons between groups are also indicated.

Interestingly, both GLP1-RA- and SGLT2i-treated patients showed a significant reduction in BMI, waist circumference, fasting blood glucose, and HbA1c at T6 compared with baseline, while control patients significantly reduced only BMI at T6 compared with T0. In particular, the improvement in BMI was more significant in the GLP1-RA group than in all the others, while the effects on fasting glycemia were significantly greater in the SGLT2i-treated group. Otherwise, during the 6-month follow-up, no significant differences were found between the three groups in terms of changes in lipid profile, e-GFR, albuminuria, and C-peptide.

**Figure 2:**
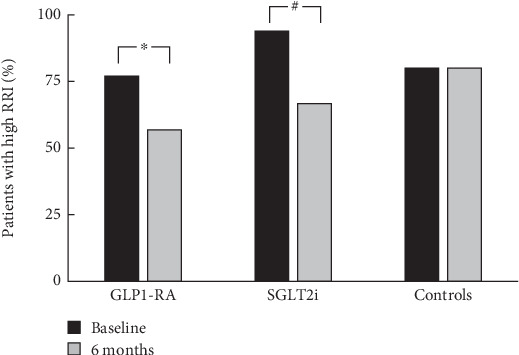
Percentage of patients with high RRI (> 0.64) at baseline (T0) and after 6 months of follow-up (T6), according to treatment. ⁣^∗^*p* < 0.001; ^#^*p* = 0.004.

As shown in Table 2, during treatment with GLP1-RA or SGLT2i, a significant decrease in RRI was observed as compared with homologous values at baseline, while in the control group RRI did not change throughout the follow-up. As a result, the improvement in RRI at T6 was significant compared with controls in both GLP1-RA- and SGLT2i-treated patients. Specifically, the percentage of patients with high RRI values (> 0.64) decreased from 77% to 56.8% and from 93.9% to 66.7% in the GLP1-RA and SGLT2i groups, respectively, while it remained almost unchanged in the group of patients receiving conventional therapy (80%) (Figure 2).

These data were confirmed using linear mixed models, correcting the analysis for major confounding factors (age, gender, diabetes duration) and changes in BMI, waist circumference, HbA1c, and e-GFR (Table 3).

A subanalysis of the GLP1-RA-treated population according to dulaglutide (*n* = 38) or semaglutide (*n* = 39) treatment revealed that the RRI improvement in the GLP1-RA group was mainly driven by the dulaglutide-treated cohort (Table 4).

**Figure 3:**
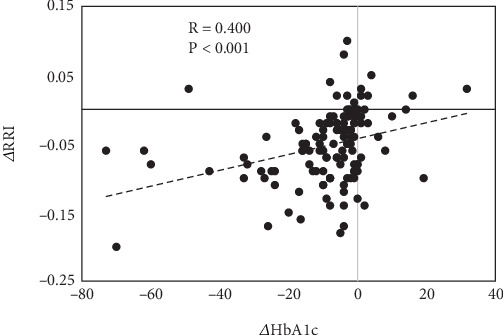
Correlation between changes in RRI and HbA1c after 6 months of follow-up in the entire study population (*n* = 145). Values are expressed as the difference between T6 and T0. Abbreviations: HbA1c: glycated hemoglobin; RRI: renal resistive index.

Finally, in the entire study population, we found that the RRI correlations with fasting blood glycemia, HbA1c, and e-GFR, observed at baseline, were still maintained after 6 months of therapy (*R* = 0.275, *p* = 0.002; *R* = 0.398, *p* < 0.001; and *R* = −0.363, *p* < 0.001, respectively). However, the correlation analysis between therapy-driven changes in all variables considered in the study showed a direct and significant correlation only between changes in RRI and changes in HbA1c (Figure 3) or fasting blood glucose (not shown).

## 4. Discussion

Chronic renal failure is universally considered one of the greatest healthcare burdens worldwide and a large proportion of patients at the time of diagnosis have T2D. Early identification and management of diabetes and diabetic nephropathy is a key goal to combat this social scourge.

The gold standard for the assessment of renal damage is biopsy; however, it is an invasive test and is burdened with high costs and major bleeding complications, as well as the risk of erroneous sampling. Hence, there is a need to develop a noninvasive, low-cost, and rapidly reproducible diagnostic tool for early detection of kidney damage.

In this context, US color Doppler evaluation of RRI may be of strategic importance for several reasons, including noninvasiveness, widespread use, low cost, repeatability, and high sensitivity. RRI is considered one of the most sensitive tools in the study of nephropathies, allowing the quantification of changes in renal plasma flow [21]. This index offers an indirect measurement of pathological changes in arteriolar resistances downstream of the point at which the sphygmic wave is sampled. However, a correct interpretation of the RRI cannot take into account only changes in renal intravascular resistances. Indeed, RRI values are also influenced by other intrarenal components, such as changes in venous and/or tubulointerstitial pressure, and by extrarenal variables, such as obstructions in the prerenal arterial circulation with post stenotic hemodynamic commitment as well as changes in heart rate or systemic arterial compliance [22].

In the current study, we firstly analyzed, in a population of patients with T2D enrolled before starting and/or intensifying antidiabetic therapy, the relationship at baseline between RRI values and clinical and biohumoral parameters. Our results confirmed the literature data on the direct relationship between RRI and age, albuminuria, and e-GFR [2, 23]. Notably, we found a significant positive relation between RRI values, triglycerides, and glycemic profile in the study population. These data might be the result of increased renal arteriolar resistance secondary to atherosclerotic disease, which is known to be closely related to T2D and hyperglycemia. Accordingly, in diabetic patients, the widespread atherosclerosis and the reduced vascular compliance may account for RRI increases even before the onset of alterations in renal function [24], supporting the idea of using RRI as an early indicator of preclinical diabetic nephropathy and prognostic marker of renal disease progression and macroalbuminuria development [4, 25, 26]. In addition, in the early stages of DKD, damage is mainly expressed at the tubulointerstitial level, resulting in increased extravascular pressure and reduced extravascular renal compliance, which are important determinants of renal resistances. In agreement with these speculations, it should be stressed that 82% of the patients enrolled in our study had high RRI values at baseline despite preserved renal function.

After 6 months of follow-up, regardless of the ongoing therapy, we confirmed the RRI correlations with fasting blood glucose, HbA1c, and e-GFR observed at baseline. However, RRI changes only correlated with changes in HbA1c or fasting blood glucose but not with e-GFR or albuminuria. In addition, no correlation was observed between changes in the glycemic profile and changes in e-GFR or albuminuria after therapy. These findings are in line with a recent report suggesting that changes in RRI may anticipate the decline in e-GFR and the onset of albuminuria in the course of diabetic nephropathy [27]. An early reduction in RRI, associated with an improvement in patients' glycemic profile, reflects an improvement in renal perfusion, tubulointerstitial commitment, and ultimately CVR.

Then, we analyzed the data from the study population, both at baseline and at T6, after subdivision into groups according to prescribed therapy. First of all, it should be pointed out that, due to the real-life nature of the study, the choice of treating patients with GLP1-RA or SGLT2i was not made in a randomized manner but mainly on the basis of therapeutic indications. Accordingly, patients with higher dysmetabolic features were preferentially treated with GLP1-RA, which explains the differences in terms of BMI, waist circumference, and HDL values observed, at baseline, between patients treated with GLP1-RA and the other groups. Following the same line of reasoning, if no contraindications were present, patients with higher levels of fasting blood glucose or HbA1c at baseline were preferentially treated with SGLT2i in order to skip the titration phase of the first month required for GLP1-RA treatment. Finally, the higher RRI values observed in patients who started SGLT2i treatment compared to those treated with GLP1-RA are in line with current diabetes management guidelines that recommend gliflozins as the first choice in patients with high CVR and cardiac and/or renal complications.

After 6 months of therapy, as previously described [28–31], patients from both the GLP1-RA and SGLT2i groups showed a significant improvement in glycemic profile, BMI, and waist circumference compared with baseline. The effect on BMI was more pronounced in GLP1-RA-treated patients, while the glycemic profile improved more in the SGLT2i group, in accordance with the primary outcome of these patients and their baseline characteristics (a significantly higher BMI and worse glycemic profile in the GLP1-RA and SGLT2i groups, respectively).

Despite the short duration of the follow-up, we found that patients undergoing therapy with GLP1-RA or SGLT2i experienced a significant reduction in RRI, with normalization of this parameter in 32% and 30% of treated patients, respectively. Importantly, this improvement did not occur in the control group and was independent of major confounding factors and changes in BMI, waist circumference, e-GFR, and HbA1c. The occurrence of a GLP1-RA- or SGLT2i-mediated decrease in RRI independently of glucose control is particularly relevant considering that, in the study population taken as a whole, changes in RRI were found to be positively correlated with changes in glycemia and HbA1c. Our finding would thus suggest a direct effect, not mediated by glucose-lowering action, of both drug classes on renal hemodynamics.

In this regard, a significant improvement in RRI after acute inhibition of SGLT2 (only 2 days of dapagliflozin treatment) has been previously described, and a direct beneficial effect on vessels, probably mediated by a reduction of oxidative stress, was therefore suggested [13]. In mouse models, empagliflozin also showed similar results [14].

To our knowledge, no other data are available on the effects of once-weekly GLP1-RAs on RRI. Although no conclusive data have yet been produced, emerging evidence suggests a direct kidney protective effect of GLP1-RAs. In experimental studies, GLP-1 has been shown to induce natriuresis and diuresis, involving an inhibition of sodium–hydrogen exchanger 3 (NHE3) located on the brush border of renal proximal tubular cells [32–34], and to reduce markers of renal RAAS activation [16], including angiotensin II and its deleterious effects on the glomerulus [35, 36]. In addition, GLP-1 modulates inflammation at several sites, including the kidneys and blood vessels, where GLP-1 receptors have been mainly revealed [32] through a decrease in cAMP-PKA-dependent ROS production [37]. Data from recent trials on the renal outcomes of injectable GLP1-RA also appear to be very promising [15, 38].

With particular reference to our study population, it should be emphasized that in the GLP1-RA-treated group, dulaglutide appeared to be more effective than semaglutide in improving RRI. Notably, this difference was not attributable to the characteristics of the enrolled patients as it was confirmed after adjusting the analysis for confounding factors and for changes in BMI, waist circumference, HbA1c, and e-GFR over the follow-up. This result was quite surprising and needs further confirmation and investigation. One possible explanation might lie in the different characteristics of dulaglutide and semaglutide degradation products. Indeed, in experimental studies, GLP-1 cleavage products have been associated with a lower expression of markers of renal tubular and tubulointerstitial damage, which supports the hypothesis of a possible nephroprotective effect independently of GLP1-R expression [16, 33, 39].

To the best of our knowledge, this study is the first real-life study to analyze the effects of chronic therapy with once-weekly GLP1-RA or SGLT2i on RRI in patients with T2D. In addition, the presence of a control group is an added value in terms of the significance of the results.

The main limitations of this study are related to the relatively small sample size and the unbalanced distribution of the three study groups, which, while not affecting the statistical analysis, displays a prevalence of GLP1-RA-treated patients. However, within this latter group, the regular proportion of patients treated with dulaglutide or semaglutide allowed us to perform an interesting comparison between the two molecules. Other potential limitations include the observational nature of the study and the variability of baseline characteristics of the study population between groups, reflecting the real-world nature of the study; single-center enrolment; and the short duration of follow-up. A larger, long-term cohort study would be appropriate to verify the effect of these treatments on renal outcomes and RRI over time.

## 5. Conclusions

Our study showed, in a population of patients with T2D, a direct relationship between RRI and patients' glycometabolic profile.

Treatment with GLP1-RA or SGLT2i significantly improved RRI independently of changes in e-GFR and albuminuria, suggesting that RRI monitoring may be a useful and simple tool to follow the evolution of DKD and the effects of therapy on renal outcomes, before changes in e-GFR and albuminuria occur.

Finally, the significant effects of GLP1-RA and SGLT2i on RRI were found to be independent of their glucose-lowering action, supporting the evidence-based benefits of these drugs on CVR and the emerging benefits on renal damage.

## Figures and Tables

**Table 1:** Baseline characteristics of study population by group membership.

**Variables**	**Study population (** **n** = 145**)**	**GLP1-RA (** **n** = 77**)**	**SGLT2i (** **n** = 40**)**	**Controls (** **n** = 28**)**
Age (years)	64.8 ± 9.5	63.6 ± 10.2	65.6 ± 7.9	66.6 ± 9.6
Diabetes duration (years)^b^	4.5 ± 10	4.0 ± 9	6.0 ± 10	1.5 ± 9
Gender [male, *n* (%)]	85 (58.6)	42 (54.6)	30 (75)	13 (46.4)
Hypertensive patients [*n* (%)]	131 (90.3)	71 (92)	35 (88)	25 (89)
BMI (kg/m^2^)^b^	31.4 ± 8.1	33.1 ± 7.8^∗^^,##^	29.1 ± 8.7	30.9 ± 6.9
Waist circumference (cm)	111.2 ± 12.9	114.6 ± 13.7^∗^^,##^	106.9 ± 12.1	107.9 ± 8.8
Fasting glycemia (mg/dL)^b^	122 ± 52	122 ± 38^##^	145.5 ± 83^∗∗^	115 ± 31
HbA1c (mmol/mol)^b^	50.0 ± 19.5	49.0 ± 17.3^##^	56.0 ± 37.3^∗∗^	47.5 ± 13.5
C-peptide (ng/mL)^b^	3.0 ± 1.7	3.0 ± 1.5	2.6 ± 3.3	2.7 ± 1.6
Total cholesterol (mg/dL)^b^	150 ± 54	150 ± 50	144 ± 56	175 ± 41
LDL cholesterol (mg/dL)^b^	79.8 ± 48	80 ± 49	72.0 ± 52	86.5 ± 48
HDL cholesterol (mg/dL)^b^	46 ± 14	45 ± 15^∗^	43.5 ± 14	52.5 ± 21
Triglycerides (mg/dL)^b^	122 ± 70	113 ± 72	127 ± 72	115 ± 70
e-GFR (mL/min/1.73 m^2^)	80.3 ± 22.3	81.9 ± 22.6	82.8 ± 20.7	72.3 ± 22.9
Albuminuria (mg/g)^b^	16.0 ± 30.2	15.0 ± 28.4	17.0 ± 40.0	17.5 ± 33.0
RRI	0.70 ± 0.06	0.69 ± 0.06^#^	0.72 ± 0.05	0.70 ± 0.07

*Note:* Data are expressed as absolute and relative (percent) frequencies for categorical variables and as mean ± SD.

Abbreviations: BMI, body mass index; e-GFR, estimated-glomerular filtration rate; HbA1c, glycated hemoglobin; HDL, high-density lipoprotein; LDL, low-density lipoprotein; RRI, renal resistive index.

^b^Median ± interquartile range for continuous variables.

⁣^∗^*p* < 0.05 versus controls.

⁣^∗∗^*p* < 0.01 versus controls.

^#^ *p* < 0.05 versus SGLT2i.

^##^ *p* < 0.01 versus SGLT2i.

**Table 2:** Changes in anthropometric and biohumoral parameters after 6 months of follow-up and statistical comparison between groups.

**Variables**	**GLP1-RA**	**SGLT2i**	**Controls**
BMI (kg/m^2^)	–2.36 ± 2.15^£,^⁣^∗∗^^,#^	−1.32 ± 1.71^£^	−0.64 ± 0.68^£^
Waist circumference (cm)	−4.24 ± 6.66^@^	−2.09 ± 5.3^@^	−0.75 ± 5.21
Fasting glycemia (mg/dL)	−20.20 ± 28.88^£,^⁣^∗∗^^,#^	−40.66 ± 54.41^£,^⁣^∗∗^	7.65 ± 33.68
HbA1c (mmol/mol)	−9.11 ± 12.21^£,^⁣^∗∗^	−13.6 ± 20.19^£,^⁣^∗∗^	−1.43 ± 9.51
C-peptide (ng/mL)	0.09 ± 1.54	−0.70 ± 1.24^@^	0.02 ± 0.84
Total cholesterol (mg/dL)	−18.24 ± 36.47	−23.42 ± 41.8^@^	−18.20 ± 35.03^@^
LDL cholesterol (mg/dL)	−14.63 ± 32.77	−17.47 ± 31.56^@^	−13.84 ± 34.70
HDL cholesterol (mg/dL)	−0.73 ± 8.50	0.17 ± 7.22	−3.39 ± 6.26^@^
Triglycerides (mg/dL)	−9.58 ± 52.71	−9.54 ± 46.27	1.63 ± 42.61
e-GFR (mL/min/1.73 m^2^)	−0.80 ± 12.80	−1.39 ± 14.29	−1.53 ± 10.45
Albuminuria (ln mg/g)	−28.20 ± 80.19	−88.34 ± 346.90	−4.8 ± 16.02
RRI	−0.05 ± 0.05^£,^⁣^∗^	0.05 ± 0.05^£,^⁣^∗∗^	0.01 ± 0.05

Abbreviations: BMI, body mass index; e-GFR, estimated-glomerular filtration rate; HbA1c, glycated hemoglobin; HDL, high-density lipoprotein; LDL, low-density lipoprotein; RRI, renal resistive index.

^@^ *p* < 0.05 versus homologous at T0.

^£^ *p* < 0.01 versus homologous at T0.

⁣^∗^*p* < 0.05 versus controls.

⁣^∗∗^*p* < 0.01 versus controls.

^#^ *p* < 0.05 versus SGLT2i.

^##^ *p* < 0.01 versus SGLT2i.

**Table 3:** Linear mixed model to estimate the changes in RRI according to antidiabetic treatment after 6 months of follow-up.

	**Adjusted model** ^**a**^		
**Treatment × time interaction**	**Effect**	**95% CI**	**p** ** value**
GLP1-RA	−0.025	−0.05 to −0.00	0.037
SGLT2i	−0.028	−0.05 to −0.00	0.041
Controls	Ref		

^a^Change in time of RRI according to antidiabetic treatment adjusted for age, gender, diabetes duration, and variations in Hba1c, BMI, waist circumference, and e-GFR.

**Table 4:** Linear mixed model to estimate the changes in RRI according to GLP1-RA treatment after 6 months of follow-up.

	**Adjusted model** ^**a**^		
**Treatment × time interaction**	**Effect**	**95% CI**	**p** ** value**
Semaglutide	−0.01	−0.04 to 0.01	0.292
Dulaglutide	−0.03	−0.06 to −0.01	0.008
Controls	Ref		

^a^Change in time of RRI according to GLP1-RA treatment adjusted for age, gender, diabetes duration, and variations in HbA1c, BMI, waist circumference, and e-GFR.

## Data Availability

The data that support the findings of this study are available from the corresponding authors upon reasonable request.

## Nomenclature

BMI: body mass index
CKD: chronic kidney disease
CV: cardiovascular
CVR: cardiovascular risk
DKD: diabetic kidney disease
DPP4-1: dipeptidyl peptidase 4 inhibitor
e-GFR: estimated glomerular filtration rate
GLP1-RA: glucagon-like peptide 1 receptor agonist
Hba1c: glycated hemoglobin
HDL: high-density lipoprotein
LDL: low-density lipoprotein
QW: once weekly
RRI: renal resistive index
SGLT2i: sodium glucose cotransporter inhibitor
T2D: Type 2 diabetes
US: ultrasonographic
